# Recombinant GMA56 and ROP17 of *Eimeria magna* conferred protection against infection by homologous species

**DOI:** 10.3389/fimmu.2022.1037949

**Published:** 2023-01-12

**Authors:** Jie Xiao, Hao Chen, Ruoyu Zheng, Jiayan Pu, Xiaobin Gu, Yue Xie, Ran He, Jing Xu, Bo Jing, Xuerong Peng, Guangyou Yang

**Affiliations:** ^1^ Department of Parasitology, College of Veterinary Medicine, Sichuan Agricultural University, Chengdu, China; ^2^ Department of Chemistry, College of Life and Basic Science, Sichuan Agricultural University, Chengdu, China

**Keywords:** *Eimeria magna*, gametocyte antigen 56, rhoptry kinase family protein 17, recombinant proteins, protective effect

## Abstract

One of the most common rabbits coccidia species, *Eimeria magna* is mainly parasitic in the ileal and jejunal epithelial cells. *E. magna* infection can affect the growth performance of rabbits or cause other secondary diseases. Traditional methods of anticoccidial treatment typically result in drug resistance and drug residue. Therefore, vaccination is a promising alternative. Gametocyte antigen 56 (GAM56) and rhoptry kinase family proteins (ROPs) are involved in oocyst wall formation and parasite invasion, respectively. A virulence factor, ROP17 contains a serine/threonine kinase catalytic domain. In this study, recombinant *E. magna* GAM56 (r*Em*GAM56) and ROP17 (r*Em*ROP17) proteins were obtained from a prokaryotic expression system and their reactogenicity was investigated with immunoblotting. To assess the potential of r*Em*GAM56 and r*Em*ROP17 as coccidiosis vaccines, New Zealand White rabbits were subcutaneously immunized with 100 μg r*Em*GAM56 (rGC group) or r*Em*ROP17 (rRC group) twice at 2-week intervals followed by homologous oocyst challenge. The rabbit serum was collected weekly to detect the specific antibody levels. The cytokine levels of pre-challenge serum were measured by enzyme-linked immunosorbent assay and the rabbits were observed and recorded post-challenge for the onset of clinical symptoms. The weight gain, oocyst output, and feed conversion ratio were calculated at the end of the experiment. The results showed that both r*Em*GAM56 and r*Em*ROP17 had good reactogenicity. The r*Em*GAM56- or r*Em*ROP17-immunized rabbits had milder clinical symptoms and feed conversion ratios of 3.27:1 and 3.37:1, respectively. The r*Em*GAM56-immunized rabbits had 81.35% body weight gain and 63.85% oocyst output reduction; the r*Em*ROP17-immunized rabbits had 79.03% body weight gain and 80.10% oocyst output reduction. The ACI of rGC and rRC groups were 162.35 and 171.03, respectively. The specific antibody levels increased rapidly after immunization. Significantly increased interleukin (IL)-2, interferon (IFN)-γ, and IL-17 levels were evident in the rGC and rRC groups (p < 0.05). The r*Em*GAM56 and r*Em*ROP17 elicited humoral and cellular responses, which protected against *E. magna* infection in rabbits. Thus, r*Em*GAM56 and r*Em*ROP17 are potential vaccine candidates against *E. magna*, and r*Em*ROP17 performed better than r*Em*GAM56.

## 1 Introduction

Rabbit coccidiosis is a highly contagious protozoan disease, and the prevalence in rabbitries is more than 90% even with the use of anticoccidials (1, 2). To date, 11 *Eimeria* spp. have been identified as the valid species of rabbit coccidiosis, among which, although *Eimeria magna* is a mildly pathogenic species but it is widely distributed in rabbitries (3, 4). *E. magna* can infect rabbits of all ages, especially juvenile rabbits (5, 6). *E. magna* parasitizes the ileum and jejunum of rabbits, causing depression, watery stool, diarrhea, reduced growth performance, reduced feed conversion efficiency, and even death of rabbits (7–9). Epidemiological investigations have suggested the high infection prevalence (17-42%) and oocyst output intensity of *E. magna* (5, 10, 11). Meanwhile, due to the development of the resistance to robenidine, some *E. magna* strains are predominant in rabbit breeding (12). Moreover, coccidiosis infection may cause other secondary diseases in subclinical conditions. Therefore, rabbit coccidiosis is responsible for large economic losses in the rabbit industry (5, 11).

Traditional coccidiosis control is heavily reliant on anticoccidials, but concerns over drug resistance and drug residue encouraged the search for new control strategies such as vaccines (13). Current research on rabbit *E. magna* vaccines has mainly focused on live attenuated vaccines (14–17). However, live anticoccidial vaccines are expensive to produce and carry the risk of virulence reversal (18). Compared with live attenuated vaccines, recombinant antigen-based subunit vaccines are high-stability and easy to mass produce, which significantly reduces the antigen production cost (19).

Secreted by rhoptry, rhoptry kinase family proteins (or rhoptry bulb proteins, ROPs) are critical in host cell invasion (20). Recently, it was reported that ROP16 (21), ROP17 (22, 23), and ROP18 (24) confer protection against apicomplexan infections. Gametocyte antigen 56 (GAM56) of chicken *E. maxima* demonstrated good antigenicity and immunogenicity (25) where the homologous gene of GAM56 was identified in *E. tenella* and *E. acervulina* (26). Chickens immunized with anti- *E. tenella* (*Et*)GAM56 antibody were protected against coccidiosis by passive immunity (27).

In the present study, the *E. magna* (*Em*)*GAM56* and *EmROP17* genes were selected and cloned based on our *E. magna* transcriptome data. Recombinant GAM56 (r*Em*GAM56) and ROP17 (r*Em*ROP17) proteins were obtained from a prokaryotic expression system. Then, we investigated the immune responses and protective effects of r*Em*GAM56 and r*Em*ROP17 against homologous challenge with *E. magna* in rabbits. To our knowledge, this is the first report of the two recombinant subunit vaccines against *E. magna*.

## 2 Materials and methods

### 2.1 Parasites and animals

The *E. magna* Chinese isolate was kindly provided by Xianyong Liu of China Agricultural University (Beijing, China) (9) and was preserved and passaged at our laboratory. Sixty coccidia-free New Zealand White rabbits (35 days old, 0.84 ± 0.108 kg, 30 females and 30 males, n = 5 females and 5 males per group) were randomly grouped then raised according to Wei et al. (28). The rabbits were housed in pairs in flame-sterilized steel cages, and a plastic partition was placed at the bottom of each cage to prevent the experimental rabbits from contacting feces. Anticoccidial drugs were discontinued 1 week before the challenge infection and pathogenic examination was performed every other day to ensure that no coccidia oocysts were detected. The rabbits were vaccinated with a bivalent vaccine against rabbit hemorrhagic disease virus and *Pasteurella multocida* when they were 30 days old.

The experimental groups were the r*Em*GAM56 (rGC) and r*Em*ROP17 (rRC) groups (r*Em*GAM56- or r*Em*ROP17-immunized and *E. magna*-challenged); the positive control groups were the unimmunized-challenged (UC, sterile phosphate-buffered saline [PBS] mock-immunized and *E. magna*-challenged), Quil-A-challenged (QC, saponin derivative Quil-A mock-immunized and *E. magna*-challenged), and rTrx-His-S-challenged (rTC) groups (recombinant pET-32a tag protein mock-immunized and *E. magna*-challenged); and the negative control unimmunized-unchallenged (UU) group (sterile PBS mock-immunized without *E. magna* challenge). The rabbits were immunized at 35 days of age, and a booster immunization was conducted 2 weeks later (49 days old); then the rabbits were challenged 2 weeks after the booster immunization (63 days old) (**Table 1**).

**Table 1:** Trial design and immune procedures.

Group	Rabbits (n)	Immunogen and dosage	Immunization ages (days old)	Immunization route	Challenge dose/age/route
Unimmunized-unchallenged (UU)	10	1 mL sterile PBS	35, 49	Neck subcutaneous injection	–
Unimmunized-challenged (UC)	10	1 mL sterile PBS	35, 49	Neck subcutaneous injection	1 × 10^5^ sporulated oocysts/63 days old/oral
Quil-A-challenged (QC)	10	1 mg Quil-A dilution in 1 mL PBS	35, 49	Neck subcutaneous injection	1 × 10^5^ sporulated oocysts/63 days old/oral
rTrx-His-S-challenged (rTC)	10	100 μg Trx-His-S tag+1 mg Quil-A dilution in 1 mL PBS	35, 49	Neck subcutaneous injection	1 × 10^5^ sporulated oocysts/63 days old/oral
r*Em*GAM56 (rGC)	10	100 μg r*Em*GAM56+1 mg Quil-A dilution in 1 mL PBS	35, 49	Neck subcutaneous injection	1 × 10^5^ sporulated oocysts/63 days old/oral
r*Em*ROP17 (rRC)	10	100 μg r*Em*ROP17+1 mg Quil-A dilution in 1 mL PBS	35, 49	Neck subcutaneous injection	1 × 10^5^ sporulated oocysts/63 days old/oral

### 2.2 *Em*GAM56 and *Em*ROP17 sequence analysis

The *Em*GAM56 and *Em*ROP17 open reading frames (ORF) and amino acid sequences were obtained using ORF Finder (https://www.ncbi.nlm.nih.gov/orffinder/). The molecular weight (MW) of the proteins was predicted with the ExPASy proteomics server (http://web.Expasy.org/protparam/). The transmembrane regions and signal peptides of the proteins were analyzed with TMHMM Server v.2.0 (http://www.cbs.dtu.dk/services/TMHMM/#opennewwindow) and the SignalP 4.1 server (http://www.cbs.dtu.dk/services/SignalP/), respectively. B cell epitopes were predicted using the Immune Epitope Database Analysis Resource (http://tools.immuneepitope.org/bcell/). Multiple sequence alignment was performed using Jalview 2.11.2.0 (29).

### 2.3 Cloning, expression, and purification

Total RNAs of *E. magna* (unsporulated oocysts, sporulated oocysts, merozoites, and gametocytes) were extracted using a commercial kit (Tiangen, Beijing, China) and the complementary DNAs (cDNAs) were synthesized (Thermo Fisher Scientific, Waltham, MA, USA). Then the resulting cDNAs were mixed and used as a template for PCR amplification.

The specific forward (F) and reverse (R) primers for *EmGAM56* and *EmROP17* were designed based on *E. magna* transcriptome data: *Em*GAM56-F 5′-CGGGATCCATGGAACCCTCTACCATTGAG-3′ and *Em*GAM56-R 5′-GCGTCGACTTAGAAAGGCATGCCTGC-3′; *Em*ROP17-F 5′-CGGGATCCATGTACAGCCTCTTACAAGGTCAC-3′ and *Em*ROP17-R 5′-GCGTCGACCTACTCTGAGCTTTTTCCTTCACT-3′, and contained *BamH*I and *Sal*I restriction enzyme sites (underlined) (Takara, Dalian, China). The purified PCR amplification products were cloned into pET-32a(+) plasmids, then the recombinant plasmids pET-32a(+)-*Em*GAM56 and pET-32a(+)-*Em*ROP17 were sequenced (Sangon, Shanghai, China) and transformed into *Escherichia coli* BL21 for protein expression (1 mM isopropyl-β-d-thiogalactoside [IPTG]). The r*Em*GAM56 and r*Em*ROP17 proteins were purified (HisTrap HP, Cytiva, Marlborough, MA, USA) then separated using 12% sodium dodecyl sulfate–polyacrylamide gel electrophoresis (SDS-PAGE). The purified fusion Trx-His-S tag protein (with no insert fragment) was cryopreserved in our laboratory.

### 2.4 Western blotting

The anti-*E. magna* positive serum and negative serum were provided by the Sichuan Agricultural University Department of Parasitology.

After 12% SDS-PAGE separation, the r*Em*GAM56 and r*Em*ROP17 were transferred onto nitrocellulose membranes (Boster, Wuhan, China). The membranes were blocked for 2 h using 5% (w/v) skimmed milk solution in Tris-buffered saline (TBS) at room temperature, then incubated overnight at 4°C with anti-*E. magna* positive serum (1:200 v/v dilution in TBS) and negative serum (1:200 v/v dilution in TBS). After four washes with TBST (TBS+0.05% Tween-20), the membranes were incubated with horseradish peroxidase-conjugated goat anti-rabbit IgG (1:2000 v/v dilution, EarthOx Life Sciences, Millbrae, CA, USA) for 2 h at room temperature. After four washes, the immunoreactive protein bands were detected using a Metal Enhanced DAB Substrate Kit (20×) (Solarbio, Beijing, China).

### 2.5 Immunization and challenge

**Figure 1:**
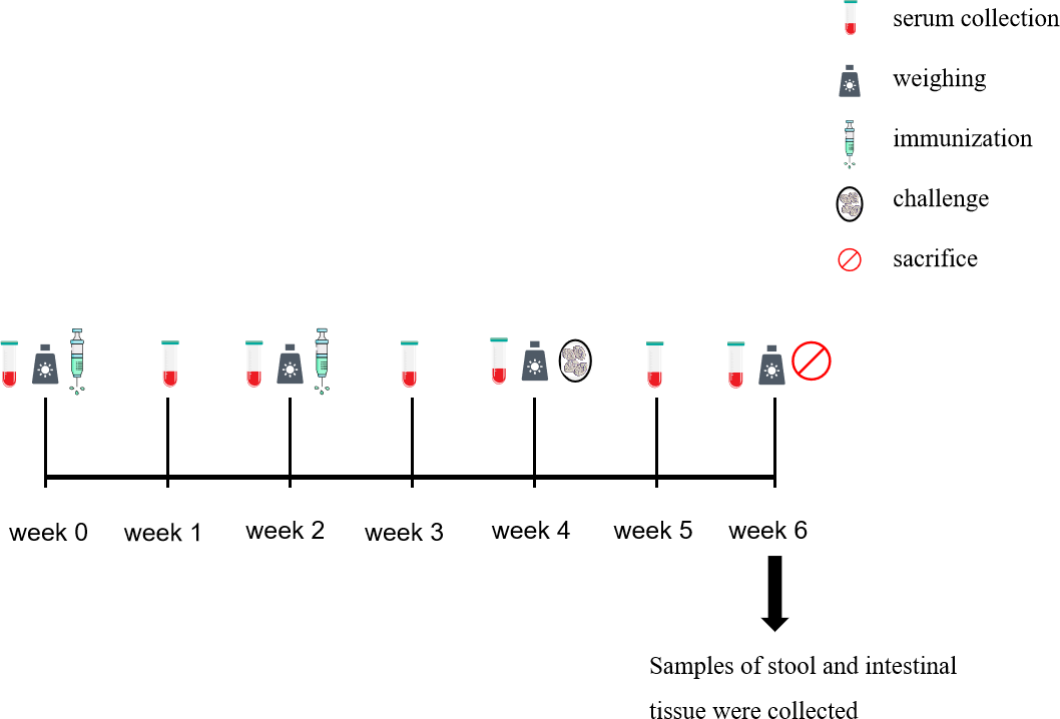
Time course of the collection of the samples.

The trial design and immune procedures are detailed in **Table 1** and **Figure 1**. The rabbits were sacrificed 2 weeks after the challenge.

### 2.6 Evaluation of protective efficacy

To evaluate the safety of the r*Em*GAM56 and r*Em*ROP17 proteins, we observed the health status and body weight gain after immunization of all experimental rabbits. The body weight of each rabbit was recorded before the first immunization, booster immunization, and challenge. The weight gain after immunization was calculated as the weight before challenge minus the weight before the first immunization to verify whether the immunization affected the weight gain of the experimental rabbits.

For protective efficacy, there is currently no specific standard for evaluating recombinant subunit vaccines against rabbit coccidiosis. In this study, after rabbits were challenged with *E. magna*, we observed whether the rabbits developed diarrhea and decreased appetite, and the protective effects were evaluated according to the following parameters among groups (n = 10 per group) (1): the survival rate (%) = the number of surviving rabbits/the initial number of rabbits × 100 (2); the body weight gain after challenge (g) = weight before sacrifice (g) − weight before challenge (g); (3) after the rabbits had been sacrificed, 2 g feces was collected from the rectum, and the amount of oocysts excreted per g feces (OPG) was calculated using the McMaster method (30), the oocyst decrease ratio (%) = (the OPG of UC group − the OPG of experimental group)/the OPG of UC group × 100 (4); feed conversion ratio = feed consumption (g)/rabbit’s mass after challenge (g), to be specific, after the challenge, the initial weight of feed for each group was recorded, and rabbits were given the same amount of feed every day; at the end of the experiment, the remaining feed of each group was weighed again (final weight), and the feed consumption of each group was obtained by subtracting the final weight from the initial weight; the feed conversion ratio was obtained by dividing the feed consumption by the total weight gain of all rabbits in each group after challenge (31); (5) ACI = (relative rate of weight gain + survival rate) − (lesion value + oocyst value), and the ACI ≥ 180 is considered good or effective, 160 ≤ ACI < 179 is considered moderately effective, ACI < 160 is considered poor effective (32–34).

### 2.7 Serum IgG level detection

The sera of all rabbits were collected pre-immunization, and then collected weekly after immunization. All serum samples were stored at −20°C.

The specific antibody levels of the immunized rabbits were evaluated using indirect enzyme-linked immunosorbent assay (ELISA) based on r*Em*GAM56 and r*Em*ROP17 (35). The optimal concentration of r*Em*GAM56 and r*Em*ROP17 was 0.94 μg/well and 1.13 μg/well, respectively. The optimal serum dilution ratio was 1:160.

### 2.8 Serum cytokine level detection

The rabbit serum interleukin (IL)-2, IL-4, IL-10, IL-17, interferon gamma (IFN-γ), and transforming growth factor beta 1 (TGF-β1) levels were detected using commercial ELISA kits (Cusabio, Wuhan, China). For each group, serum of six rabbits were randomly selected.

### 2.9 Statistical analysis

Differences among the groups were assessed using one-way analysis of variance (ANOVA) with IBM SPSS Statistics 22.0 (IBM, Armonk, NY, USA). GraphPad Prism 8.0.2 (GraphPad Software Inc., La Jolla, CA, USA) was used to produce all the graphs. P < 0.05 and < 0.01 were considered significant and extremely significant, respectively.

## 3 Results

### 3.1 *Em*GAM56 and *Em*ROP17 sequence features

The *EmGAM56* gene (GenBank accession number: OM451230) ORF was 1371 bp (encoding a protein with a predicted MW of 51 kDa) while that of the *EmROP17* gene (GenBank accession number: OM451229) was 1725 bp (encoding a protein with a predicted MW of 63 kDa). Neither *EmGAM56* nor *EmROP17* contain a transmembrane region, but the signal peptides were predicted at 1–20 and 1–22 amino acids, respectively. The *EmGAM56* and *EmROP17* target fragment sizes without signal peptides were 1314 bp and 1662 bp, respectively.

**Figure 2:**
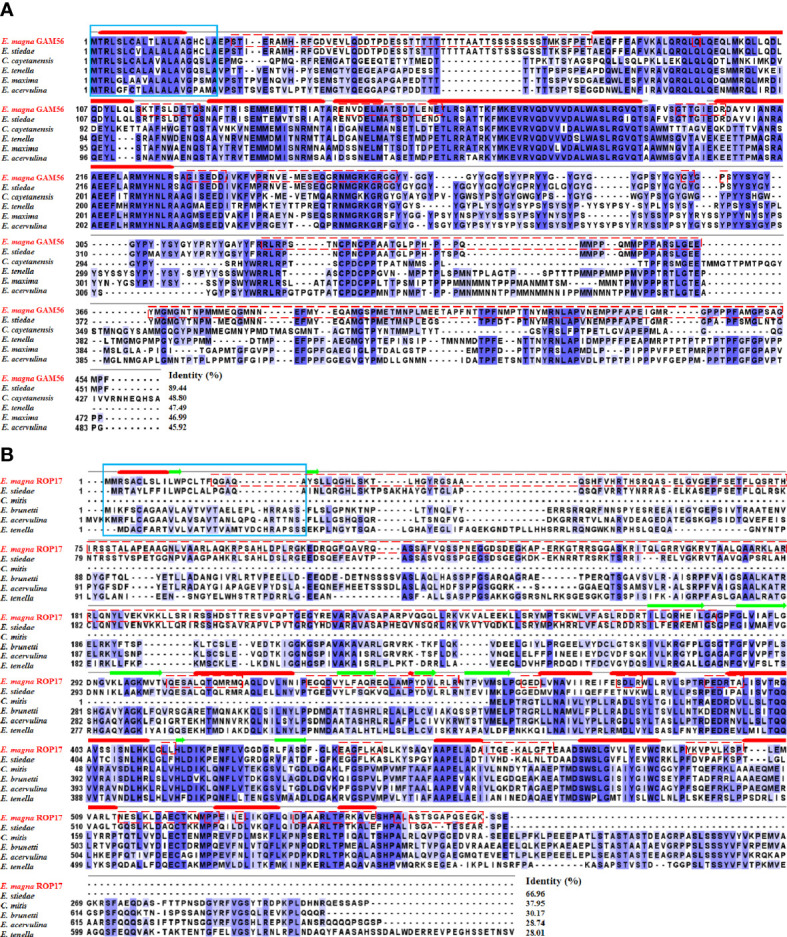
Multiple sequence alignment of GAM56 and ROP17 from different species. **(A)** Multiple sequence alignment of *Em*GAM56 with GAM56 proteins from *E stiedae* (GenBank accession number: OL622034), *Cyclospora cayetanensis* (UniProt: A0A1D3D9G4), *E tenella* (UniProt: U6KUA4), *E maxima* (UniProt: U6M5G7), and *E acervulina* (UniProt: U6GGM3). **(B)** Multiple sequence alignment of *Em*ROP17 with ROP17 proteins from *E stiedae* (GenBank accession number: OM451231), *E mitis* (UniProt: U6KAV7), *E brunetti* (UniProt: U6LD89), *E acervulina* (UniProt: U6GVC3), and *E tenella* (UniProt: U6KG78). Blue shading indicates conserved residues. Dashed red outlines represent B cell epitopes. The signal peptides are marked with a solid blue outline. The thick solid red lines indicate predicted alpha helix, and the green arrows indicate predicted beta sheet.

Multiple sequence alignment revealed that the *Em*GAM56 amino acid sequences had high variability but shared high homology with rabbit *E. stiedae* (89.44%). The *Em*ROP17 amino acid sequences shared 66.96% identity with *E. stiedae* ROP17 proteins and 28.01~37.95% identity with that of other apicomplexans (**Figure 2**).

### 3.2 Expression, purification, and western blotting

**Figure 3:**
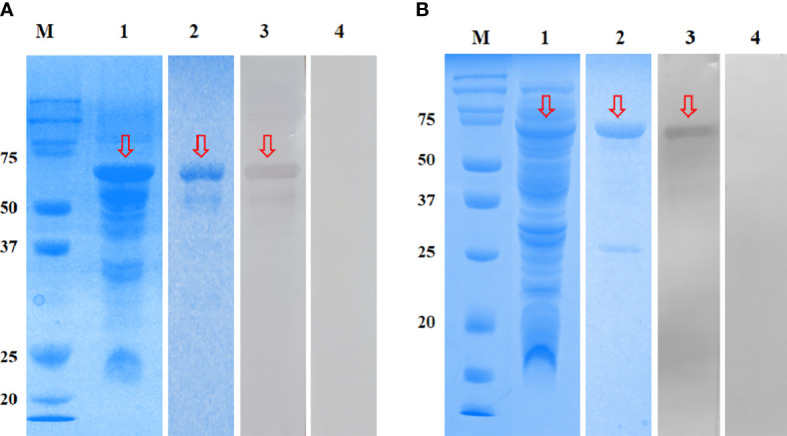
SDS-PAGE and Western blotting analysis of r*Em*GAM56 **(A)** and r*Em*ROP17 **(B)**. Lane M: Protein molecular weight markers; lane 1: crude extracts expressed by BL21 (DE3); lane 2: purified recombinant proteins; lane 3: purified recombinant proteins incubated with anti-*E. magna* positive serum; lane 4: purified recombinant proteins incubated with negative serum from coccidia-free rabbits. Arrows indicate the bands of interest.

The r*Em*GAM56 (~48 kDa) and r*Em*ROP17 (~54 kDa) were expressed in the supernatant of *E. coli* BL21 cells after IPTG induction (**Figure 3**, lane 1). The MW of the recombinant proteins included the ~20 kDa fusion tag protein encoded by pET-32a(+) plasmid. After HisTrap HP affinity column purification, the recombinant proteins were separated using 12% SDS-PAGE (**Figure 3**, lane 2).

The r*Em*GAM56 and r*Em*ROP17 were reacted with anti-*E. magna* positive serum and specific bands were observed on the NC membranes (**Figure 3**, lane 3), while the coccidia-free rabbit serum yielded no specific bands (**Figure 3**, lane 4). These results indicated that both r*Em*GAM56 and r*Em*ROP17 had strong reactogenicity.

### 3.3 Protective efficacy of r*Em*GAM56 and r*Em*ROP17

No statistically significant differences were observed for weight gain after immunization among the six groups (p *>* 0.05) (**Table 2**), nor were obvious adverse reactions observed in the immunized rabbits. This result suggested that r*Em*GAM56 and r*Em*ROP17 had good safety at the experimental doses.

**Table 2:** Protective effects of r*Em*GAM56 and r*Em*ROP17 against *E. magna* infection under different evaluation indicators.

Group	Average body weight gain after immunization (g)	Average body weight gain after challenge (g)	Relative body weight gain rate (%)	Oocyst shedding per rabbit (×10^4^/g)	Oocyst decrease ratio (%)	Feed conversion ratio	Survival rate (%)	Mean lesion scores	ACI
Unimmunized-unchallenged (UU)	785.70 ± 134.06^a^	526.10 ± 92.57^a^	100	0^a^	–	2.66:1	100	0^a^	200
Unimmunized-challenged (UC)	772.50 ± 140.56^a^	327.50 ± 236.01^b^	62.25	3.16 ± 1.01^b^	0	4.27:1	100	1.60 ± 0.70^b^	106.25
Quil-A-challenged (QC)	781.00 ± 118.18^a^	337.00 ± 109.55^b^	64.06	3.12 ± 1.14^b^	1.14	4.15:1	100	1.30 ± 0.48^bcd^	111.06
rTrx-His-S-challenged (rTC)	794.00 ± 121.28^a^	355.56 ± 183.79^b^	67.58	3.41 ± 1.17^b^	-7.99	3.94:1	90 (9/10)	1.40 ± 0.97^bc^	103.58
r*Em*GAM56 (rGC)	788.50 ± 114.31^a^	428.00 ± 122.71^ab^	81.35	1.14 ± 0.49^c^	63.85	3.27:1	100	0.90 ± 0.74^cd^	162.35
r*Em*ROP17 (rRC)	774.50 ± 121.41^a^	415.80 ± 74.30^ab^	79.03	0.63 ± 0.25^c^	80.10	3.37:1	100	0.70 ± 0.67^d^	171.03

The data are presented as the mean ± standard deviation. In each column, significant differences between the data are indicated with different superscript letters (a, b, c, d; ANOVA, p < 0.05) and data marked with the same superscripted letter are not significantly different (p > 0.05).

**Figure 4:**
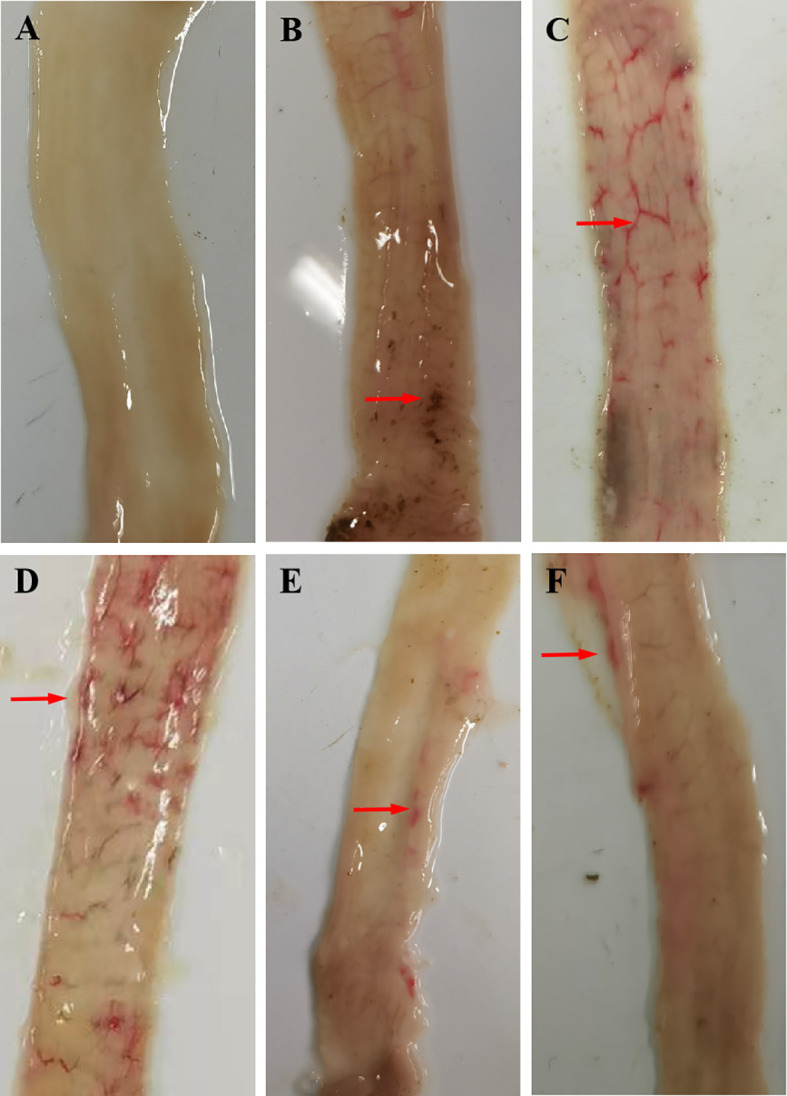
Gross postmortem examination. **(A)** UU, **(B)** UC, **(C)** QC, **(D)** rTC, **(E)** rGC, and **(F)** rRC groups.

Rabbits in the positive control groups demonstrated a slight loss of appetite and weight loss 2 weeks after the challenge; only a few rabbits had diarrhea, most of which manifested as soft unformed feces. No obvious clinical symptoms developed in the rGC and rRC groups, and a minority of rabbits had soft unformed feces. Gross postmortem examination of the positive control groups revealed obvious hemorrhagic spots in the ileum and lower jejunum while the immunized groups had few or no hemorrhagic spots (**Figure 4**).

Compared with the UC group, the relative body weight gain rate of the rGC and rRC groups was 81.35% and 79.03%, respectively (p *>* 0.05). In addition, the rabbits immunized with r*Em*GAM56 (63.85% oocyst reduction ratio) and r*Em*ROP17 (80.10% oocyst reduction ratio) had significantly lower oocyst output (p < 0.05).

The rGC (3.27:1) and rRC (3.37:1) groups had better feed conversion ratios in comparison with the UC (4.27:1), QC (4.15:1), and rTC (3.94:1) positive control groups. In addition, the results of ACI indicated that r*Em*GAM56 and r*Em*ROP17 could provide moderately effective protection.

### 3.4 IgG responses against r*Em*GAM56 and r*Em*ROP17

**Figure 5:**
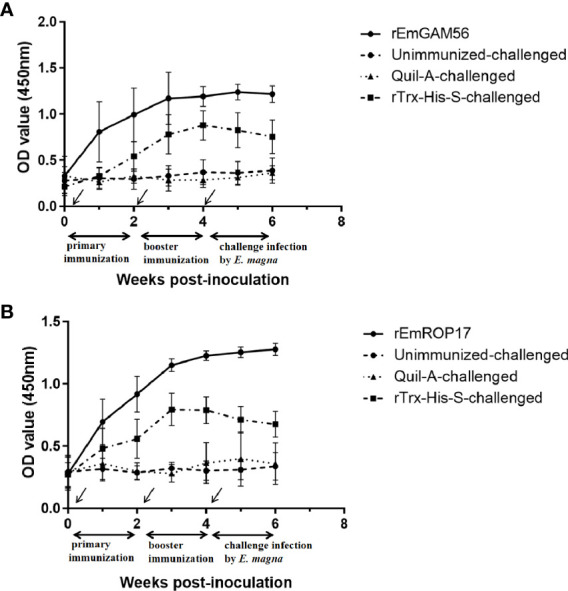
The changes in serum anti-r*Em*GAM56 **(A)** and r*Em*ROP17 **(B)** IgG levels after the first immunization (week 0), booster immunization (week 2), and challenge with *E magna* (week 4).

The serum specific IgG levels of the rGC and rRC groups increased significantly after immunization (**Figure 5**). The specific IgG levels of the rGC group peaked at week 3 but decreased at week 5 while that of the rRC group continued to increase after the challenge. The rTC group also exhibited increased antibody levels, indicating that the inclusion of the Trx-His-S tag in the r*Em*GAM56 and r*Em*ROP17 proteins increased the antibody levels. Nevertheless, the rTC group had lower antibody levels than the rGC and rRC groups.

### 3.5 r*Em*GAM56- and r*Em*ROP17-induced serum cytokine levels

**Figure 6:**
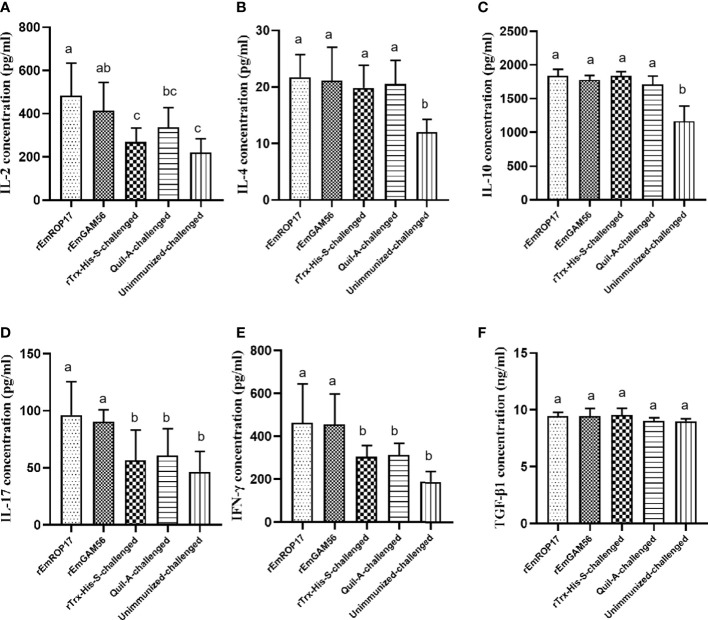
The serum IL-2 **(A)**, IL-4 **(B)**, IL-10 **(C)**, IL-17 **(D)**, IFN-γ **(E)**, and TGF-β1 **(F)** levels 2 weeks after booster vaccination. Different superscript letters (a, b) indicate a significant difference (p < 0.05). The same superscript letters indicate no significant difference (p > 0.05).

The serum cytokine levels were estimated 2 weeks after the booster vaccination. In the rGC group, serum IL-17 and IFN-γ levels were significantly increased (p < 0.05) and serum IL-2 levels were significantly higher (p < 0.05) than that of the UC and rTC groups, but there was no significant difference with the QC group (p *>* 0.05). The rRC group had significantly increased serum IL-2, IL-17, and IFN-γ levels (p < 0.05). There was no significant difference in the serum TGF-β1 levels among the groups (**Figure 6**).

## 4 Discussion

Rabbit coccidiosis is a common and highly contagious parasitic protozoan disease (3, 11). Anticoccidials are the main control method of rabbit coccidiosis but can be accompanied by drug resistance and drug residue. Previous work on anticoccidial vaccines predominantly focused on live vaccines, specifically the precocious lines. Several precocious lines of rabbit *Eimeria* spp. have been successfully selected and characterized so far, such as that for *E. magna*, and immunogenicity studies have been conducted (8). Mohamed et al. (17) reported 97% oocyst output reduction in rabbits following vaccination with 3500 oocysts from an *E. magna* precocious line. However, live anticoccidial vaccines are expensive to produce and carry the risk of virulence reversal. Therefore, recombinant subunit vaccines are a promising vaccination strategy as they are easier to mass-produce than live vaccines and have a longer shelf life (19). Some studies that explored the protective effects of recombinant subunit vaccines against chicken coccidiosis yielded good results (36, 37). For rabbit coccidiosis, early studies have reported that the soluble antigens in the bile and coproantigen from *E. stiedae*-infected rabbits could induce protection against *E. stiedae* infection (38–40). Meanwhile, our previous studies found that rabbits immunized with the recombinant protein-based subunit vaccines also displayed good protective effects against *E*. *stiedae* or *E*. *magna* infection (41, 42). These studies showed the feasibility of developing vaccines using the immunodominant antigens of rabbit coccidia.

Produced by *Eimeria* spp. gametocytes, GAM56 is involved in oocyst wall formation (43, 44). The *E. maxima* GAM56 antigen is one of the main components of CoxAbic^®^, where chickens immunized with its antibody had 78% oocyst output reduction (27, 45). Moreover, chickens immunized with a DNA vaccine carrying *Emaxi*GAM56 had 89.7% relative body weight gain and 53.7% oocyst output reduction (46). Here, the rabbits immunized with r*Em*GAM56 had significantly reduced oocyst output (63.85% reduction rate, p < 0.05) and up to 81.35% relative body weight gain.

ROPs are important in Apicomplexa host invasion (20). Some ROPs (such as ROP16, ROP17, ROP18) are serine/threonine kinases that act as virulence factors (47, 48). Several ROPs have been tested for immunogenicity, including ROP17 (49). BALB/c mice immunized with recombinant *Toxoplasma gondii* ROP17 protein received apparent protection against chronic infection, and the liver and brain parasite burdens were reduced by 59.17% and 49.08%, respectively; in immunized mice, survival increased by 50% against lethal infection (22). Liu et al. (23) identified the ROP17 of chicken *E. tenella*, where challenge experiments revealed that immunization with r*Et*ROP17 protein significantly reduced oocyst output, and the body weight gain was up to 84.14%. In the present study, r*Em*ROP17-immunized rabbits had 79.03% body weight gain and 80.10% oocyst output reduction. The results demonstrated that both the r*Em*GAM56 and r*Em*ROP17 proteins conferred protection against *E. magna* infection and that r*Em*ROP17 performed better for reducing oocyst output.

Cytokines play a role in fighting coccidiosis. A Th1 immune response marker, IFN-γ is considered a key factor for preventing coccidiosis (50). Chicken IFN-γ inhibited *Eimeria* sporozoite development *in vitro* and its recombinant protein also exerted an anticoccidial effect (51, 52). IL-2 exerted an anticoccidial effect by inducing T cell proliferation and increasing CD8^+^ and CD4^+^ T cell ratios (50). IFN-γ and IL-2 also acted as anticoccidial vaccine adjuvants to enhance the immune response to vaccine antigens (53). Combining vaccine antigens with IFN-γ or IL-2 further improved the anticoccidial index of chickens (54–56). In the present study, the IL-2 and IFN-γ levels of the immunized rabbits were significantly increased post-vaccination (p < 0.05), indicating that r*Em*GAM56 and r*Em*ROP17 stimulated Th1-type immune responses. We also observed increased IL-17 levels in the rGC and rRC groups (p < 0.05). IL-17 is important in responses against parasite infection (57). Wild-type mice treated with IL-17A neutralizing antibody had prolonged survival after being challenged with *T. gondii* (58). Ding et al. (55) reported that simultaneous immunization with recombinant 3-1E protein and IL-17 gene *in ovo* enhanced the immune protection against *E. maxima* infection in chickens. Geriletu et al. (59) also reported that the IL-17 gene enhanced the anticoccidial effect of antigens.

In the present study, both the r*Em*GAM56 and r*Em*ROP17 proteins exerted moderately protective effects against *E. magna* infection, and r*Em*ROP17 performed better for reducing oocyst output. Immunized with r*Em*GAM56 and r*Em*ROP17 could induce humoral immunity in the rabbits, and the specific IgG was significantly increased. Recent research has proven that antibodies are involved in the occurrence of *Eimeria* infection (60, 61), where antigen-specific antibodies inhibited adhesion to host cells (62). Moreover, it was shown that there was an excellent correlation between antibody titer and protection (63). As an obligate intracellular parasite, *Eimeria* spp. has a complex life cycle including the asexual and sexual replicative stages, and the asexual replicative stages (sporozoites and merozoites) lead to the most damage to the intestinal tissues (64, 65). ROPs play an important role during the early stages of host invasion, and ROP17 is expressed in the sporozoites and merozoites (23, 66, 67). Gametocyte antigens are involved in oocyst wall formation in the later sexual replicative stage (43, 44). Therefore, we speculated that the anti-r*Em*ROP17 antibody might interact with sporozoites or merozoites, and inhibit the invasion of intestinal epithelial cells by them to alleviate intestinal damage; while the anti-r*Em*GAM56 antibody mainly plays the role of inhibiting the oocyst wall formation, but at this time, the parasites have completed asexual replicative stages, causing irreversible damage to the intestinal tissues, this may lead to a better protective effect of r*Em*ROP17. Meanwhile, the high IFN-γ, IL-2, and IL-17 levels in the immunized rabbits further inhibited the intracellular infection of *E. magna*. Together, these effects might eventually lead to significant differences in oocyst output and body weight gain.

## 5 Conclusions

The r*Em*GAM56 and r*Em*ROP17 proteins conferred protective immunity against *E. magna* infection in rabbits. The relative body weight gain of the r*Em*GAM56- and r*Em*ROP17-immunized rabbits was 81.35% and 79.03%, respectively, and the oocyst output reduction rate was 63.85% and 80.10%, respectively. The r*Em*ROP17 performed better in reducing oocyst output. The r*Em*GAM56 and r*Em*ROP17 proteins elicited cellular and humoral immune responses and are potential vaccine candidates against *E. magna*.

## Data availability statement

The datasets presented in this study can be found in online repositories. The names of the repository/repositories and accession number(s) can be found below: https://www.ncbi.nlm.nih.gov/genbank/, OM451230 https://www.ncbi.nlm.nih.gov/genbank/, OM451229.

## Ethics statement

The animal study was reviewed and approved by The Sichuan Agricultural University Animal Care and Use Committee of reviewed and approved the animal study protocol (SYXK 2019–189). All animal procedures used in this study were performed in accordance with the Guide for the Care and Use of Laboratory Animals (National Research Council, Bethesda, MD, USA) and the recommendations of the Animal Research: Reporting of *In Vivo* Experiments (ARRIVE) guidelines (http://www.nc3rs.org.uk/arrive-guidelines). All applicable institutional and national guidelines for the care and use of animals were followed.

## Author contributions

JXi participated in the design of the study, fed the experimental animals, and performed the experiments, statistical analysis, and manuscript writing. HC fed the experimental animals and performed the experiments. RZ, JP, and BJ contributed to the sample collection. GY participated in the design of the study. XG, YX, RH, JXu, XP, and GY assisted in the study design. All authors read and approved the final manuscript.
