# Supplementary material for: Recombinant GMA56 and ROP17 of Eimeria magna conferred protection against infection by homologous species
Source: Front Immunol. 2023 Jan 12;13:1037949. doi: 10.3389/fimmu.2022.1037949 (PMC9879601; doi:10.3389/fimmu.2022.1037949)
Supplement: Supplementary file 1 [file DataSheet_1.docx]

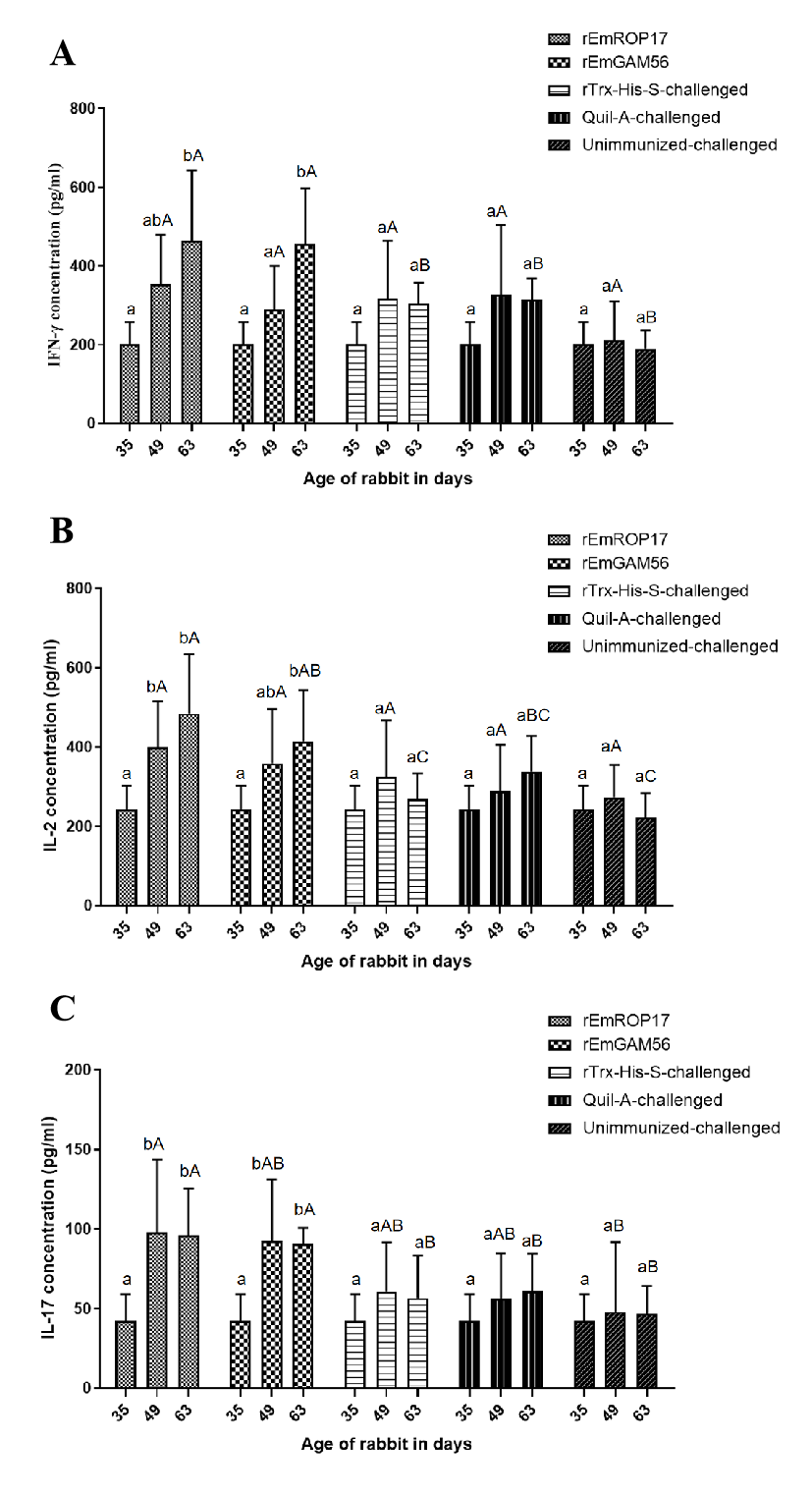


**Supplementary Figure** The IFN-γ **(A)**, IL-2 **(B)**, IL-17 **(C)** response to immunization. Different superscript in small letters (a, b; ANOVA, p < 0.05) indicate a significant difference in interleukin within a group, and different superscript in capital letters (A, B, C; ANOVA, p < 0.05) indicate a significant difference between groups for the same day.
